# Insecticide susceptibility status of *Anopheles gambiae* mosquitoes and the effect of pre-exposure to a piperonyl butoxide (PBO) synergist on resistance to deltamethrin in northern Namibia

**DOI:** 10.1186/s12936-024-04898-y

**Published:** 2024-03-14

**Authors:** Rosalia N. Joseph, Tabeth Mwema, Seth J. Eiseb, Deodatus V. Maliti, Munyaradzi Tambo, Iitula Iitula, Lydia Eloff, Ophilia Lukubwe, Cara Smith-Gueye, Élodie A. Vajda, Allison Tatarsky, Stark T. Katokele, Petrina N. Uusiku, Dennis Walusimbi, Sheila B. Ogoma, Davis R. Mumbengegwi, Neil F. Lobo

**Affiliations:** 1 University of Namibia (UNAM), Windhoek, Namibia; 2 Clinton Health Access Initiative (CHAI), Boston, MA USA; 3 Ministry of Health and Social Services (MoHSS), Windhoek, Namibia; 4 Malaria Elimination Initiative, University of California, san francisco, San Francisco, USA; 5 Namibia University of Science and Technology, Windhoek, Namibia; 6 University of Notre Dame, Notre Dame, IN USA; 7 Washington University School of Medicine, St. Louis, MO USA

**Keywords:** *Anophele*s *gambiae *sensu lato, Insecticide resistance, Pyrethroid, PBO synergist

## Abstract

**Background:**

Pyrethroid-based indoor residual spraying (IRS) and long-lasting insecticidal nets (LLINs) have been employed as key vector control measures against malaria in Namibia. However, pyrethroid resistance in *Anopheles* mosquitoes may compromise the efficacy of these interventions. To address this challenge, the World Health Organization (WHO) recommends the use of piperonyl butoxide (PBO) LLINs in areas where pyrethroid resistance is confirmed to be mediated by mixed function oxidase (MFO).

**Methods:**

This study assessed the susceptibility of *Anopheles gambiae *sensu lato (*s.l*.) mosquitoes to WHO tube bioassays with 4% DDT and 0.05% deltamethrin insecticides. Additionally, the study explored the effect of piperonyl butoxide (PBO) synergist by sequentially exposing mosquitoes to deltamethrin (0.05%) alone, PBO (4%) + deltamethrin (0.05%), and PBO alone. The *Anopheles* mosquitoes were further identified morphologically and molecularly.

**Results:**

The findings revealed that *An*. *gambiae *sensu stricto (*s*.*s*.) (62%) was more prevalent than *Anopheles arabiensis* (38%). The WHO tube bioassays confirmed resistance to deltamethrin 0.05% in the Oshikoto, Kunene, and Kavango West regions, with mortality rates of 79, 86, and 67%, respectively. In contrast, *An. arabiensis* displayed resistance to deltamethrin 0.05% in Oshikoto (82% mortality) and reduced susceptibility in Kavango West (96% mortality). Notably, there was reduced susceptibility to DDT 4% in both *An. gambiae s.s*. and *An. arabiensis* from the Kavango West region. Subsequently, a subsample from PBO synergist assays in 2020 demonstrated a high proportion of *An. arabiensis* in Oshana (84.4%) and Oshikoto (73.6%), and 0.42% of *Anopheles quadriannulatus* in Oshana. Non-amplifiers were also present (15.2% in Oshana; 26.4% in Oshikoto). Deltamethrin resistance with less than 95% mortality, was consistently observed in *An. gambiae s.l.* populations across all sites in both 2020 and 2021. Following pre-exposure to the PBO synergist, susceptibility to deltamethrin was fully restored with 100.0% mortality at all sites in 2020 and 2021.

**Conclusions:**

Pyrethroid resistance has been identified in *An. gambiae s.s.* and *An. arabiensis* in the Kavango West, Kunene, and Oshikoto regions, indicating potential challenges for pyrethroid-based IRS and LLINs. Consequently, the data highlights the promise of pyrethroid-PBO LLINs in addressing resistance issues in the region.

## Background

Malaria continues to pose a significant public health challenge in nine northern regions of Namibia: Zambezi, Kavango East, Kavango West, Kunene, Ohangwena, Oshikoto, Omusati, Oshana, and Otjozondjupa [1, 2]. In contrast, the southern regions of Namibia remain generally malaria-free [1, 2]. Namibia has relied on indoor residual spraying (IRS) as the primary vector control intervention since 1965 [1]. Initially, dichlorodiphenyltrichloroethane (DDT) was the insecticide of choice, primarily applied to traditional mud and thatch structures. However, in 2005, the country shifted its approach to using deltamethrin, a pyrethroid insecticide, targeting modern cement structures [3]. This transition reflects Namibia's adaptability in response to changing malaria dynamics. The introduction of long-lasting insecticidal nets (LLINs) with pyrethroids to complement IRS in the mid-2000s marked another significant stride in malaria control [4, 5]. LLINs have demonstrated their high effectiveness in reducing malaria transmission throughout sub-Saharan Africa [6, 7].

Entomological surveillance in various regions has shown that approximately 30% of mosquito exposure occurs indoors [8]. This finding underscores the potential effectiveness of LLINs and IRS in reducing malaria transmission. The distribution of LLINs in Namibia has been substantial, with a target of one net for every two individuals at risk of malaria, following World Health Organization (WHO) guidelines [9]. The impact of LLINs on malaria transmission in sub-Saharan Africa, with significant reductions in malaria cases [6, 7], justifies ongoing efforts by the National Malaria Control Programmes (NMCPs) to increase LLIN ownership and usage in Africa [10–12]. Despite significant headway, malaria remains a public health problem in Namibia, with 13,633 reported cases in 2020 [12]. It is possible that pyrethroid resistance reduced the efficacy of IRS and LLINs. Using pyrethroid insecticides in IRS and LLINs in Africa, despite widespread pyrethroid resistance among *Anopheles* mosquito populations, may contribute to the increase in malaria cases [1]. Pyrethroid resistance has been detected in at least one malaria vector in more than two-thirds of the sites tested, with the highest in the WHO regions of Africa and the Eastern Mediterranean [13]. The WHO recommends annual monitoring of insecticide resistance in major malaria vectors to guide vector control strategies [13]. Local entomological surveillance of vector susceptibility to insecticides is crucial, as recommended by the WHO [14].

This study was conducted as part of the annual entomological surveillance of the National Vector-borne Disease Control Programme (NVDCP) and aimed to assess the species-specific susceptibility of *Anopheles gambiae *sensu lato (*s.l*.) to DDT and deltamethrin insecticides. In addition to evaluating susceptibility to traditional insecticides, the NVDCP has been actively exploring innovative approaches to malaria vector control. Notably, the NVDCP has considered the distribution of novel pyrethroid piperonyl butoxide (PBO) LLINs, which have been shown to increase the mortality of malaria vectors with metabolic resistance involving monooxygenases [8]. These PBO LLINs combine a pyrethroid insecticide with the synergist PBO, increasing their effectiveness against pyrethroid-resistant *Anopheles* mosquitoes [15]. The PBO synergist inhibits metabolic enzymes, such as mixed function oxidases (MFO) of the cytochrome P450 family, preventing the sequestration of pyrethroids by mosquitoes before they become toxic [11, 16].

## Methods

### Study sites

**Fig. 1:**
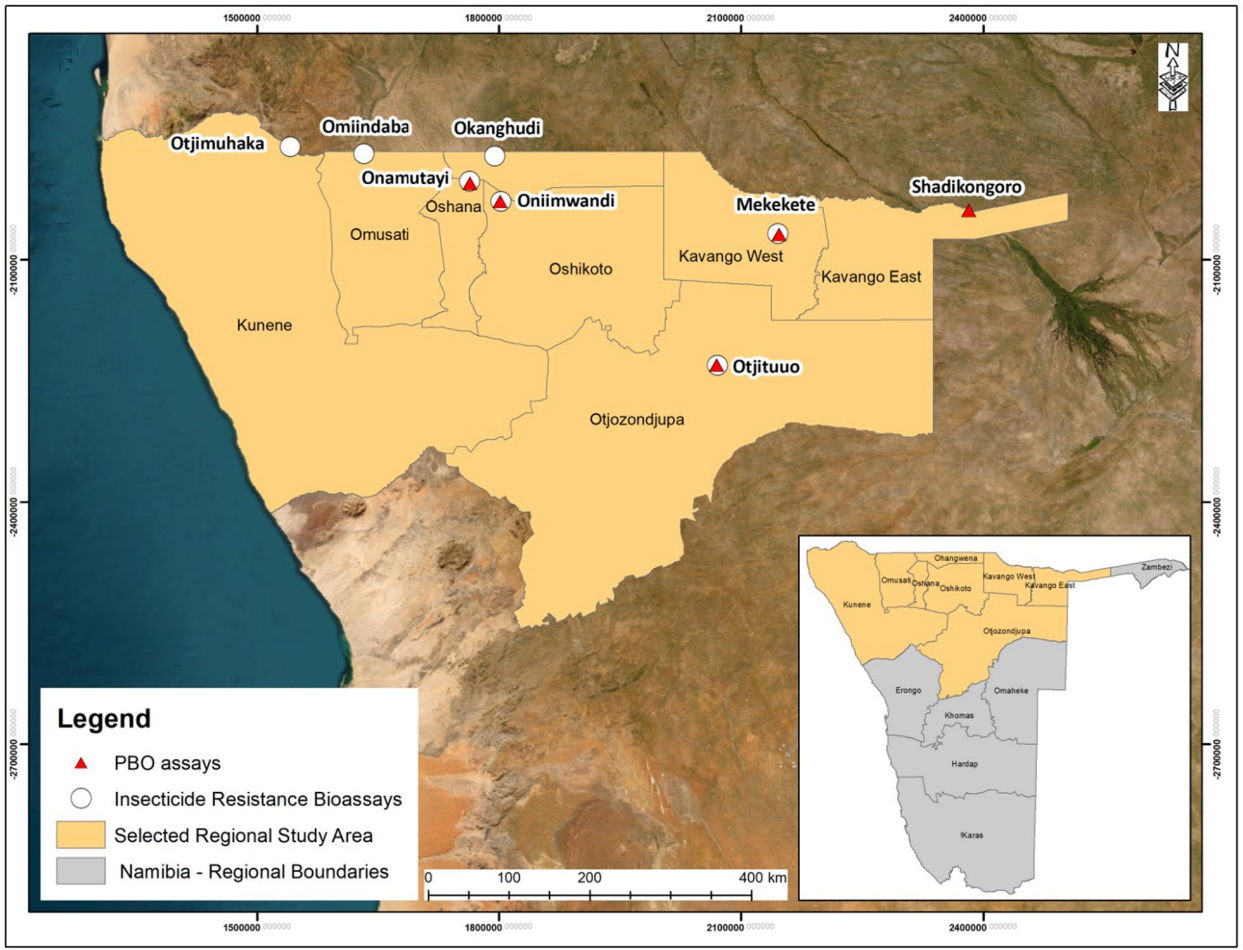
Sentinel sites (and regions) in Namibia where larvae were collected for the insecticide susceptibility test and PBO assays

The insecticide susceptibility study in 2018 was conducted at sentinel sites in seven of the nine malaria-endemic regions. The seven regions (sentinel village sites in brackets) that were included in this study were Oshana (Onamutai), Oshikoto (Oniimwandi), Otjozondjupa (Otjituuo), Ohangwena (Okanghudi), Kunene (Otjimuhaka), Omusati (Omiindaba), and Kavango West (Mukekete) (Fig. 1). Thereafter, PBO synergist assays were conducted in sentinel sites from six malaria-endemic regions, which included the Oshana region (Onamutai), Oshikoto (Oniimwandi), Otjozondjupa (Otjituuo), Kavango West (Mukekete), Kavango East (Shadikongoro), and Zambezi (Sibbinda) (Fig. 1). A sentinel site in each region was a malaria hotspot determined by the NVDCP based on malaria risk maps generated from malaria surveillance data. Each sentinel site included a sampling area with a radius of approximately 10 km^2^ from the central location of the village.

### Collection and rearing of larvae

Larvae and pupae were collected from the study sentinel sites from March to April 2018 for insecticide assays. For PBO synergist assays, larvae and pupae were only collected from sites in the Oshikoto and Oshana regions during the peak malaria season (March to May 2020). The larval collections were disrupted from March to April 2020 due to restrictions imposed by the COVID-19 pandemic. Additionally, collections from sites in the Oshikoto, Otjozondjupa, Kavango West, Kavango East, and Zambezi regions were not possible from March to April 2021, once again due to the ongoing COVID-19 restrictions. Larval sampling was performed by using larval scoops dipped at the edges of breeding sites [17, 18]. Specific sampling sites in each region were chosen by the NVDCP based on malaria risk maps generated from malaria surveillance data. Each sentinel site included a sampling area of approximately a 10 km radius from the central location of the village. *Anopheles* mosquito larvae and pupae were reared to adulthood at the NVDCP insectary in Oshakati. The larvae were fed a powdered mixture of dog biscuits and yeast, while the adults were fed on 10% sugar solution ad libitum. Insecticide susceptible *An. arabiensis* mosquitoes (KGB strain, Witwatersrand University) were used for the control assays. The temperature and humidity within the insectary were maintained between 28 °C and 29 °C and 70 and 80%, respectively. Female *Anopheles* mosquitoes aged 3–5 days that had never had a blood meal were used for the assays.

### WHO insecticide susceptibility bioassays

Using WHO-recommended procedures [13, 17], a complete test included four replicates using 20 adult wild-caught specimens per tube and two control replicates each using 20 susceptible *Anopheles arabiensis* mosquitoes per tube. In total, 1680 female *Anopheles* were exposed to the WHO bioassays, while 280 mosquitoes were used as controls. Adult wild female mosquitoes (collected as larvae), 3–5 days old, were exposed to DDT (4%) and deltamethrin (0.05%) insecticide-impregnated papers in WHO tubes for one hour. Two control tubes, each lined with paper impregnated with silicone oil, were run concurrently with the insecticide assays testing. Following exposure, mosquitoes were provided with a 10% sugar solution and maintained at 28 °C to 29 °C with 70–80% humidity. Mosquito mortality was observed and recorded after 24 h. The observed mortality of the test sample was calculated by summing the number of dead mosquitoes across all exposure tubes and then expressing this as a percentage of the total number of exposed mosquitoes. Mosquitoes were stored separately on silica gel for molecular analysis.

### PBO synergist assays

WHO Whatman papers impregnated with the PBO synergist were used to perform the synergist assay. PBO-synergist assays were conducted using WHO-recommended procedures [13, 17]. A complete test included four holding tubes (each with 20 wild caught female mosquitoes) and five exposure tubes. The sample size was ~ 60 mosquitoes for sentinel sites and 20 *An. arabiensis* for the control replicates. A total of 120 female mosquitoes were used for the synergist-insecticide monitoring study from the Oshana (Onamutayi village) and Oshikoto regions (Oniimwandi and Omandongo village) in 2020. In 2021, 300 wild caught female mosquitoes were used from Oshikoto (Oniimwandi), Otjozondjupa (Otjituuo), Kavango West (Mukekete), Kavango East (Shadikongoro) and Zambezi (Sibbinda). For each sentinel site, a total of only 60 mosquitoes were exposed to the PBO synergist.

The mosquitoes were subjected to exposed in three WHO tubes under distinct conditions: (i) a dosage of 4% “PBO only”, (ii) pre-exposed to 4% “PBO” for one hour followed by exposure to 0.05% Deltamethrin for an additional hour (PBO + Deltamethrin), and (iii) exposure to 0.05% “Deltamethrin only”. Concurrently with the PBO assays, a control assay was conducted using a tube lined with paper impregnated with silicone oil. Test mosquitoes were maintained at 28°–29 °C and a relative humidity range of 70–80% during exposure and holding periods. Test mosquitoes were provided with 10% sugar solution ad libitum during the holding periods. Mosquito mortality was observed and recorded after 24 h. The observed mortality of the test sample was calculated by summing the number of dead mosquitoes across all exposure tubes and then expressing this as a percentage of the total number of exposed mosquitoes. This calculation was performed for each insecticide at each site.

### Molecular identification of *Anopheles gambiae*

Dead and live mosquitoes were separated and labelled at the end of each assay. All mosquitoes were morphologically identified using keys [19] as *An. gambiae s.l.* All samples for 2018 and a subsample from 2020 underwent molecular species identification using polymerase chain reaction (PCR) diagnostic [20].

### Analysis

WHO guidelines [13] were used to interpret the susceptibility status of *An. gambiae s.l.* mosquitoes after 24 h of exposure to insecticides. A mean mortality of > 98% indicated susceptibility to the insecticide, while a mean mortality between 90 and 98% indicated reduced susceptibility to the insecticide, and a mean mortality of < 90% indicated resistance to the insecticide. The Kruskal–Wallis test was used to compare mosquito mortality between the sentinel sites using the Statistical Package for the Social Sciences (SPSS) version 27 software. A Pearson's chi-square test was used to compare the *Anopheles* mosquito species composition between the regions in the R statistical package 4.1.2. WHO guidelines [13] were used to interpret the effect of the PBO synergist on mosquitoes. “If the mean mortality in the “insecticide only” samples is ≥ 90%, the effect of PBO cannot be reliably assessed. If the mean mortality in the “insecticide only” samples is < 90%, the effect of PBO can be interpreted according to the following criteria:

- Complete restoration of susceptibility (mitigation of resistance) by pre-exposure to PBO (i.e., ≥ 98% mean mortality in the “PBO followed by insecticide” samples) implies that a monooxygenase-based resistance mechanism fully accounts for the expression of the resistant phenotype in the test population.
- Partial restoration of susceptibility by pre-exposure to PBO (i.e., mean mortality in the “PBO followed by insecticide” samples is greater than mean mortality in the “insecticide only” samples but < 98%) implies that a monooxygenase-based resistance mechanism only partially accounts for expression of the resistant phenotype and that other resistance mechanisms are likely to be present in the test population.

All insecticide resistance analyses are presented for *An. gambiae s.l*., with the exclusion of non-amplifiers.

### *Anopheles* species compositions

Morphologically, all species in the bioassays were identified as *An. gambiae s.l.* Of the mosquitoes tested for insecticide susceptibility (n = 1680), 1582 (94%) were successfully amplified using the *An. gambiae s.l.* diagnostic assay [20]. *Anopheles gambiae *sensu stricto (*s.s*.) made up the majority of amplified specimens (62%), and *An. arabiensis* came in second (38%). Non-amplified samples (n = 98) were not included in downstream analyses.

Due to capacity and funding limitations, only 72% (n = 309) of the PBO synergist assay samples from 2020 were subjected to molecular analysis. Approximately 84.3% (n = 200) of the samples molecularly identified from the Oshana region (n = 237) were *An. arabiensis,* 0.42% (n = 1) *Anopheles quadriannulatus,* and 15.2% (n = 36) did not amplify with PCR (non-amplifiers). From the Oshikoto region, 73.7% (n = 53) were *An*. *arabiensis*, and 26.4% (n = 19) were non-amplifiers. Samples from 2021 were not molecularly analysed. All results are presented for *An. gambiae s.l*. Overall, *An. arabiensis* (n = 253, 81.8%) was the dominant vector recorded.

**Fig. 2:**
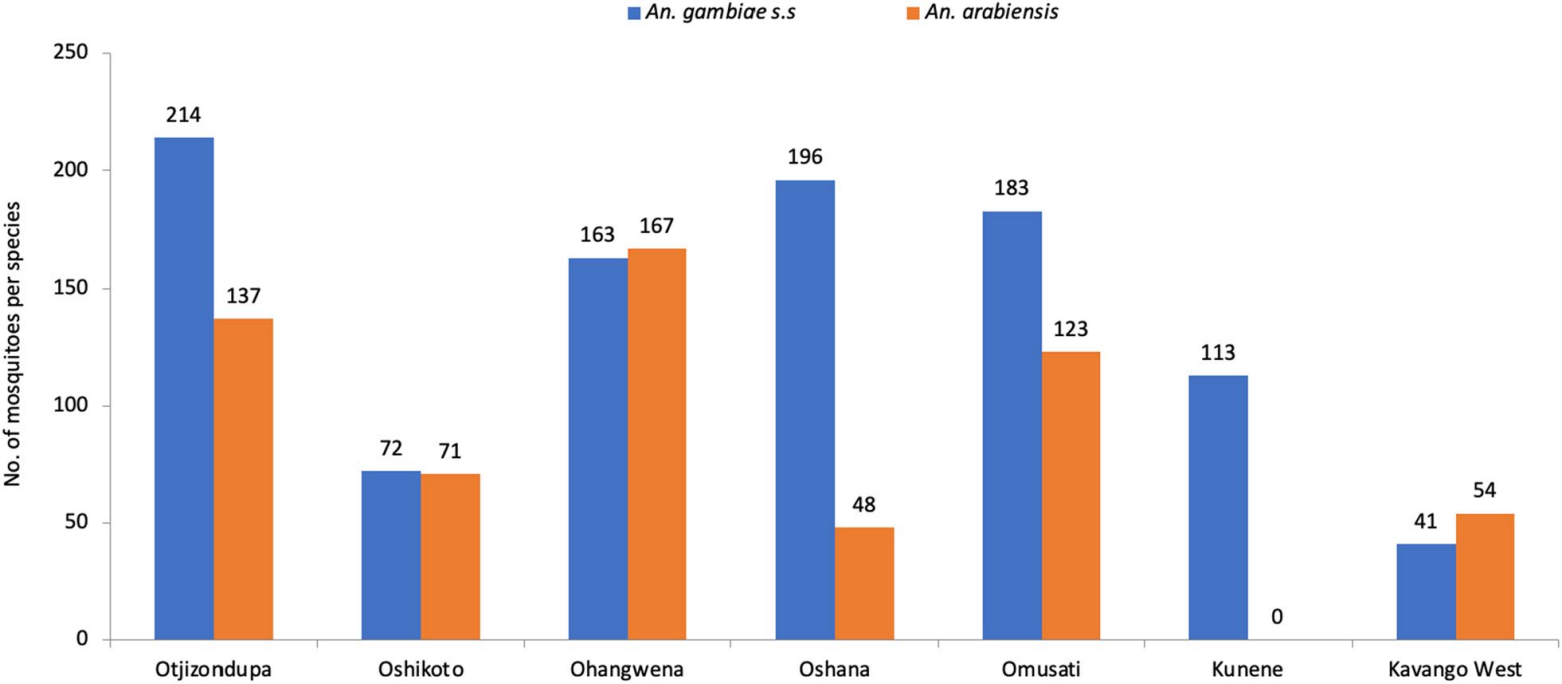
Relative composition of *An. gambiae* s.s. and *An. arabiensis* across sentinel sites in northern Namibia

A higher proportion of *An. gambiae* s*.s.* mosquitoes were sampled at most sites: 60.9% (n = 214) in Otjozondjupa, 80.3% (n = 196) in Oshana, 59.8% (n = 183) in Omusati, and 100% (n = 113) in Kunene. Approximately equal proportions of *An. gambiae s.s.* and *An. arabiensis* were observed in Oshikoto (50.3% (n = 72) and 49.7% (n = 71), respectively). Similarly, in Ohangwena, the proportions were 49.4% (n = 163) for *An. gambiae s.s.* and 50.6% (n = 167) for *An. arabiensis*, respectively), while in Kavango West, a higher proportion of *An. arabiensis* (56.8%; n = 54) was observed (Fig. 2). There was a significant difference in the species composition of the *An. gambiae* species complex between the regions (Pearson χ^2^, DF = 6, P = 0.001).

### WHO Susceptibility bioassays

After conducting the WHO tube bioassays, molecular analyses were performed on a total of 1582 female *An. gambiae s.l.* mosquitoes. Of those, 980 were *An. gambiae s.s.* and 602 were *An. arabiensis* mosquitoes.

The results demonstrate that *An. gambiae s.s.* was resistant to deltamethrin 0.05% in Oshikoto, Kunene, and Kavango West (79, 86, and 67%, respectively) and that *An. gambiae s.s.* was less sensitive to deltamethrin in Omusati (97%) and Ohangwena (94%), as shown in Table 1. *Anopheles gambiae s.s.* showed reduced susceptibility to DDT in Kavango West (91%), while they showed full susceptibility (100%) in the rest of the regions. *Anopheles arabiensis* showed resistance to deltamethrin 0.05% in the Oshikoto region (82%), reduced susceptibility in Kavango West (96%), and full susceptibility (100%) in the rest of the regions. There is a reduced susceptibility of *An. arabiensis* to DDT 4% in the Kavango West (96%) region, whereas *An. arabiensis* vectors from the other regions showed full susceptibility (100%). A Kruskal-Wallis H test was performed for these data from 2018 and suggests that there is a difference in mean mortality by DDT and deltamethrin between the regions (P = 0.008; DF = 8).

**Table 1:** *Anopheles gambiae *sensu stricto and *Anopheles arabiensis* insecticide susceptibility results using the WHO tubes in 2018

Species	Insecticide	Percentage mortality (number of mosquitoes in assay)						
		Oshana	Omusati	Ohangwena	Oshikoto	Otjozondjupa	Kunene	Kavango west
An. gambiae s.s	DDT (4%)	100% (n = 33)^a^	100% (n = 38)^a^	100% (n = 39)^a^	100% (n = 16)^a^	100% (n = 68)^a^	100% (n = 71)^a^	91% (n = 23)^a^
	Deltamethrin (0.05%)	100% (n = 55)^a^	97% (n = 74)^b^	94% (n = 88)^b^	79% (n = 56)^c^	99% (n = 96)^a^	86% (n = 42)^c^	67% (n = 18)^c^
An. arabiensis	DDT (4%)	100% (n = 14)^a^	100% (n = 36)^a^	100% (n = 14)^a^	100% (n = 38)^a^	100% (n = 89)^a^	(n = 0)^d^	97% (n = 31)^b^
	Deltamethrin (0.05%)	100% (n = 24)^a^	100% (n = 55)^a^	100% (n = 54)^a^	82% (n = 33)^c^	100% (n = 48)^a^	(n = 0)^d^	96% (n = 24)^b^

^a^Susceptible: mortality rate of ≥ 98%

^b^Reduced susceptibility: mortality rate of 91%–97%

^c^Confirmed resistance: mortality rate of ≤ 90%

^d^Species not found

### PBO-synergist assay

**Fig. 3:**
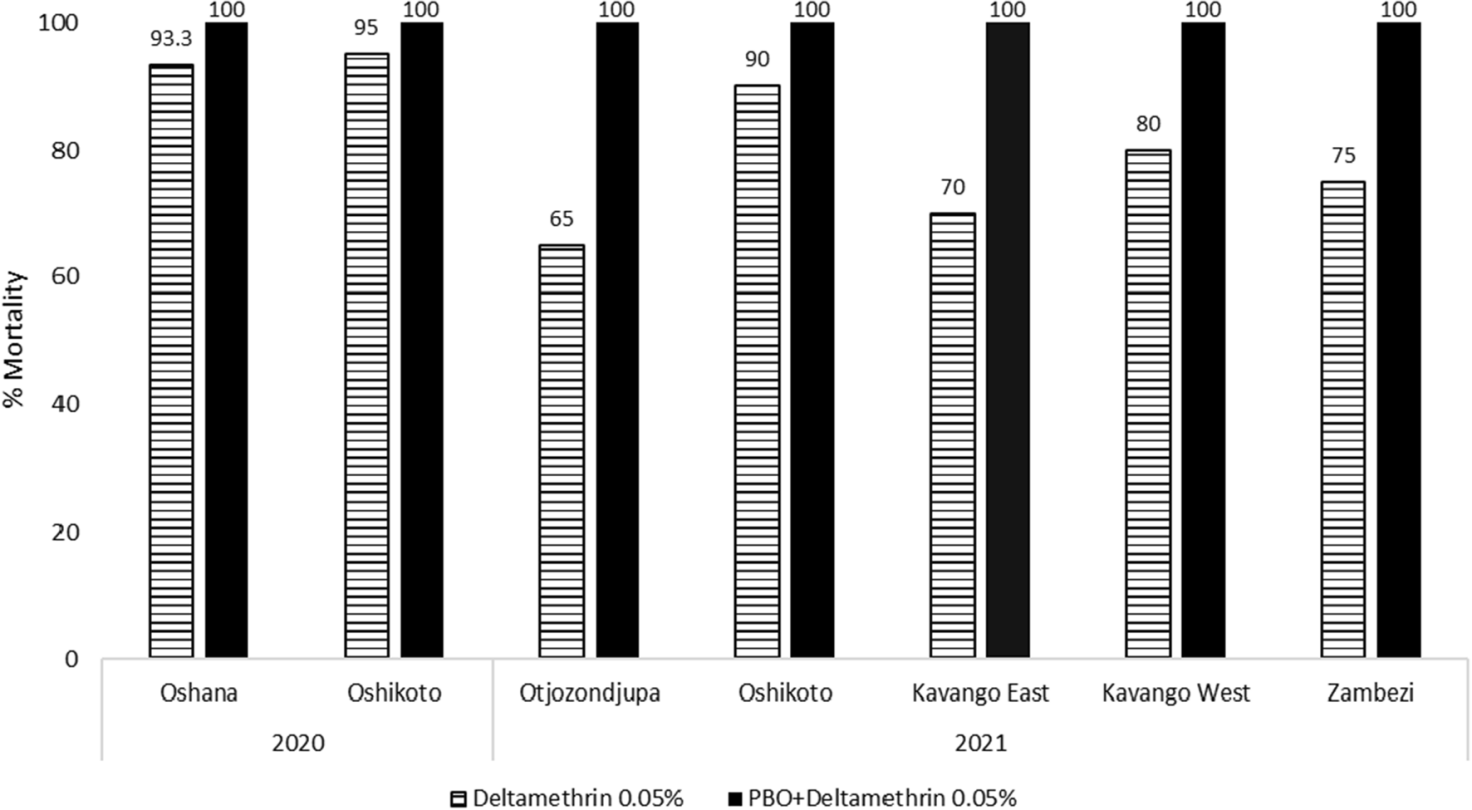
Percentage mortality of *An. gambiae* s.l. mosquitoes exposed to PBO synergist + deltamethrin, deltamethrin, and PBO across sites surveyed for 2020 and 2021

In 2020, deltamethrin (0.05%) alone induced 93.3 and 95.0% mortality in Oshana and Oshikoto, respectively. After pre-exposure to the PBO synergist (4%) followed by exposure to deltamethrin (0.05%), the resistance status of mosquitoes in the Oshana and Oshikoto regions was completely restored to 100% susceptibility (Fig. 3). Mortality after pre-exposure to the PBO synergist increased by 6% in Oshana (from 93.3 to 100%) and by 5% in Oshikoto (from 95 to 100%). In 2021, deltamethrin (0.05%) alone induced 95.0, 90.0, 70.0, 80.0, and 75.0% mortality in Otjozondjupa, Oshikoto, Kavango East, Kavango West, and Zambezi, respectively. After pre-exposure to the PBO synergist (4%) followed by exposure to deltamethrin (0.05%), the resistance status of mosquitoes was completely restored to 100% susceptibility in all five regions (Otjozondjupa, Oshikoto, Kavango East, Kavango West, and Zambezi). The results also indicated that deltamethrin resistance in the Oshikoto region increased by 5% from 2020 to 2021 (from 95.0 to 90.0% mortality) (Fig. 3).

## Discussion

The results of this study provide a comprehensive view of the *Anopheles* species compositions at the end of the rainy season, shedding light on critical implications for malaria control in Namibia. Notably, the analysis revealed that the *Anopheles* population consisted predominantly of *Anopheles gambiae s.l.*, with *An. arabiensis* accounting for 38% and *An. gambiae s.s.* for 62% of the specimens. Remarkably, the presence of *An. gambiae s.s*. in Namibia, thought to have disappeared in the mid-2000s [21], suggests a resurgence of this vector species, potentially driven by factors such as low-quality indoor residual spraying (IRS) and inadequate coverage. This resurgence carries significant implications for the region’s malaria control efforts.

*Anopheles gambiae s.s.* has also been reported as the primary vector in neighbouring Angola [22] and, when compared to *Anopheles funestus*, is the most prevalent vector in Zimbabwe [23]. This underscores its potential for cross-border re-invasion and its adaptability to drier regions, such as Otjozondjupa, challenging previous assumptions [5, 24]. Consequently, scaling up IRS with effective insecticides, considering insecticide resistance, becomes imperative, particularly in malaria hotspots, with a recommended coverage of 85% or more [21]. In contrast, *An. arabiensis*, typically associated with lower rainfall floodplains, was unexpectedly abundant in the wetter and more humid Kavango West and Ohangwena regions, mirroring findings by Kamwi [21]. This uneven distribution of *An. gambiae s.l.* species underscores the influence of microenvironmental factors on vector presence [5]. Notably, the confirmation of both *An. gambiae s.s.* and *An. arabiensis* as a primary vector in Namibia, linked to malaria transmission in some parts of Africa [25, 26], raises concerns about year-round transmission with seasonal peaks [1, 12].

Moreover, parallel studies highlight the presence of other potential malaria vectors, such as *Anopheles coustani s.l*., *Anopheles squamosus*, *Anopheles pharoensis*, and *An. funestus* [27]. The non-amplifiers in this study point to the presence of non-*An. gambiae s.l.* samples—and probably include these species. Their inclusion in insecticide resistance tests, particularly for *An. funestus*, is essential for a comprehensive understanding of the impact of insecticides. Regarding the insecticide resistance aspect, the data reveal concerning trends. Some WHO insecticide resistance bioassays did not meet the required mosquito density due to operational constraints. Nevertheless, the results indicate reduced mortality (< 90%), signifying resistance to deltamethrin (0.05%) in both *An. gambiae s.s*. and *An. arabiensis* in specific regions. This resistance extends to the extensively used insecticide deltamethrin, mirroring trends observed in other countries [28, 29]. Insecticide resistance emerges as a significant threat to malaria control efforts [30].

Furthermore, the findings underscore the need for a shift in Namibia's IRS policy, advocating for insecticides other than pyrethroids and DDT. Given the increasing pyrethroid resistance in areas with high agricultural activity, such as Oshikoto and Kavango West, the role of agricultural insecticide uses in exerting selective pressure on *Anopheles* mosquitoes becomes evident [14]. Resistance to DDT is also observed, possibly due to knockdown resistance (*kdr*)-based cross-resistance between pyrethroids and DDT [31]. Such resistance can disrupt control efforts, leading to sporadic malaria outbreaks. Preserving the efficacy of pyrethroid-impregnated bed nets, a widely used vector control method, becomes crucial. The introduction of mosquito nets incorporating the PBO synergist, designed to counter metabolic resistance to pyrethroids [15, 32, 33], presents a potential solution. Notably, this study points to MFO-detoxifying enzymes as a likely resistance mechanism in *An. gambiae s.l.* populations [15], aligning with findings from Ghana. PBO synergist exposure successfully restored susceptibility to pyrethroids, demonstrating its operational potential.

However, it is important to note that PBO LLINs should ideally increase mortality by at least 10% [16], which was not consistently achieved across all regions. Nonetheless, the complete restoration of susceptibility in all tested sites with a ≥ 10% increase in mortality confirms the involvement of MFO enzymes in deltamethrin resistance [16].

Further research and replication of studies on PBO synergist are needed to ascertain their impact, as the efficacy of PBO LLINs may vary depending on local resistance levels and other resistance mechanisms [16, 33–35]. Overall, this study contributes to the growing body of evidence supporting the potential of PBO LLINs for malaria control in areas where resistance, although not assessed for intensity, has been confirmed. While specific resistance intensity assays were not performed in this study, the findings align with the broader context of resistance challenges, suggesting that PBO LLINs could be valuable tools in regions with reported moderate-to-high pyrethroid resistance.

## Conclusion

In conclusion, this study underscores the pressing issue of insecticide resistance in malaria vectors in Namibia. Pyrethroid resistance is evident in two important vector species and is more pronounced in the predominant species, *Anopheles. gambiae s.s*., which is anthropophagic and endophilic. This finding raises concerns about the potential impact on the effectiveness of indoor residual spraying (IRS) and long-lasting insecticidal nets (LLINs), both critical components of malaria control efforts. It also poses a challenge to achieving the goal of malaria elimination. Addressing insecticide resistance demands a multifaceted approach, such as integrated vector management (IVM) strategies that encompass a range of interventions, including IRS, LLINs, larviciding, and personal protective tools such as repellents. Such a comprehensive approach can help reduce human-vector contact and disrupt the vector's life cycle at multiple stages [36]. One unexpected finding was the predominance of *An. gambiae s.s.* over *An. arabiensis* in arid regions, suggesting that the bionomics of Namibian *Anopheles* vectors of malaria may vary significantly across different ecological niches. This calls for further site-specific research to better understand the dynamics of malaria vectors in these dry areas.

Nonetheless, the study also revealed a promising avenue for overcoming pyrethroid resistance through pre-exposure to PBO synergists. This preexposure restored susceptibility, leading to 100% mortality among pyrethroid-resistant *An. gambiae* s*.l.* mosquitoes. This finding suggests the potential efficacy of pyrethroid-impregnated PBO LLINs in Namibia. It is important to note that PBO synergistic tests were conducted on wild *An. gambiae s.l.* complex mosquitoes, collected as larvae. Therefore, it will be important to complete the PCR assays to distinguish species-specific insecticide susceptibility to provide valuable insights into the dynamics of resistance among different vector species. Continued surveillance and monitoring of resistance, along with the inclusion of other vectors such as *An. funestus*, are vital components of Namibia’s malaria elimination strategy.

## Data Availability

The data supporting this study's findings is available from the Ministry of Health and Social Services, Namibia. However, restrictions apply to the availability of this data, as it was used under license for the current study and is not publicly available. Nonetheless, the authors can provide the data upon reasonable request and with permission from the MoHSS.

## Abbreviations

CHAI: Clinton health access initiative
DDT: Dichlorodiphenyltrichloroethane
EHP: Environmental health practitioners
IRS: Indoor residual spraying
IVM: Integrated vector management
*kdr*: Knockdown resistance
LLIN: Long-lasting insecticidal net
MFO: Mixed-function oxidases
MoHSS: Ministry of health and social services
NMCP: National malaria control programme
NVDCP: National vector-borne disease control programme
PBO: Piperonyl butoxide
PCR: Polymerase chain reaction
RDT: Rapid diagnostic test
*s.l.*: Sensu lato
*s..*: Sensu stricto
UNAM: University of Namibia
WHO: World Health Organization
